# Dual harm among patients attending a mental health unit in Uganda: a hospital based retrospective study

**DOI:** 10.1186/s12888-024-05560-2

**Published:** 2024-02-22

**Authors:** Alain Favina, Joan Abaatyo, Mark Mohan Kaggwa

**Affiliations:** 1 Department of Psychiatry, Faculty of Medicine, Mbarara University of Science and Technology, Mbarara, Uganda; 2 Department of Psychiatry and Behavioral Neurosciences, McMaster University, Hamilton, Canada

**Keywords:** Dual harm, Self-harm, Aggression, Male, Mental health, Uganda

## Abstract

**Background:**

Dual harm encompasses the complex interplay of the co-occurrence of self-harm and aggression. Individuals with dual harm may display a more hazardous pattern of harmful behaviors like homicide-suicide compared to people with sole harm. This study aimed to examine the presence of dual harm among general psychiatry inpatients in a mental health unit in Uganda.

**Methods:**

A retrospective chart review of 3098 inpatients from January 2018 to December 2021. Dual harm reported experience at admission was based on experiences of self-harm with harm to people or property or both. Logistic regression assessed the association between dual harm and sociodemographics and clinical characteristics.

**Results:**

A total of 29 (1%) patients experienced dual harm, with five having experienced self-harm with both harm to others and property, 23 with harm to people, and one with harm to property. Dual harm was statistically significantly associated with the male gender at bivariate analysis. However, there were no statistically significant factors associated with dual harm at multivariate analysis or sensitivity analysis with the specific types of dual harm.

**Conclusion:**

General psychiatry inpatients in Uganda experience dual harm before admission at lower prevalence than in previous literature. However, no investigated sociodemographic and clinical factors could explain these experiences. Further studies looking at dual harm are warranted to understand these unfortunate experiences with serious consequences among patients in Uganda.

## Introduction

Dual harm encompasses the complex interplay of the co-occurrence of self-harm and aggression [1]. In addition to violent crimes, aggression can take many forms, such as verbal, psychological, physical, or toward other people or property [2]. On the other hand self-harm can take the form of suicidality or (non-suicidal) self-injury (NSSI) [1]. Individuals suffering from mental disorders often exhibit behaviors that pose risks to both themselves and the people or objects around them [3–5]. These people may have severe emotional turmoil, excessive rage, or a deep sense of hopelessness, which may manifest as violent outbursts or destructive behavior toward other people or property [2, 6]. Additionally, these individuals may turn their aggression inwards, resorting to self-harming behaviors as a way to cope with their emotional pain [2, 7]. Contrasted to those who engage in self-harm alone or aggression alone (“sole-harm”), individuals who dual-harm may display a more hazardous pattern of harmful behaviors like homicide-suicide [1, 8–10].AudioVolumeMute

A systematic review by Shafti et al., (2023) estimated that the overall prevalence of dual harm is above 20%, based on studies involving 15,094 individuals from different countries [10]. Previous research have also found that dual harm was more common among institutionalized individuals, especially male patients admitted to psychiatric hospitals [1, 11, 12] or correctional facilities [13–18]. However, dual harm was not limited to these populations, as it was also reported among patients in general psychiatry settings, university students, children and adolescents, homeless individuals, among others [19]. Despite extensive research on dual harm in psychiatry, there is a gap in the literature regarding the burden of dual harm among individuals in Africa.

The phenomenon of experiencing dual harm is associated with numerous multifaceted factors, which can be categorized into three domains: childhood experiences, biological factors, psychological factors and psychiatric diagnosis. (i) Childhood experiences: The family environment in which a person grows up can have a lasting impact on their propensity for dual harm. Studies have shown that negative childhood experiences within the family, such as abuse, household dysfunction, harsh discipline, and early neglect, are associated with aggression and dual harm [20–22]. Other family-related risk factors for dual harm include, poverty, family criminality, and educational underachievement [1, 23, 24]. Moreover, adverse childhood events, such as child abuse and parental death, increase the likelihood of dual harm [16]. (ii) Biological factors: Some biological factors, such as younger age and male sex, are related to dual harm [1, 2, 11]. Additionally, a person’s personality style may predispose them to harmful behaviors when biological elements and unfavorable environmental conditions coincide [9]. (iii) Psychological factors and mental health conditions: A person’s mental state and coping skills can also affect their tendency for dual harm. Individuals who experience depression or other mental health issues, use drugs or alcohol, observe self-harm, or are victims of violence are more likely to transition from sole harm at an early age to dual harm later in life [2]. Among adolescents, dual harm is linked to harsh parenting style, substance use, low school engagement, and low self-control [25, 26]. Furthermore, adolescents with high levels of anxiety and depressive symptoms are more prone to engage in dual harm [25]. Substance abuse disorders and personality disorders (anti-social, borderline, and schizotypal) are significantly associated with dual harm in psychiatric patients [1]. Mood disorders are also documented to increase the risk of dual harm [16]. Additionally, adolescents who develop psychosis before the age of 18 years are more likely to engage in dual harm [25]. Trait impulsivity also predicts dual harm, especially in individuals with borderline personality disorder [27].

Dual harm poses a significant risk to individuals’ lives and well-being [9]. It has public health implications, as it contributes to increased healthcare utilization, emergency department visits, and psychiatric hospitalizations [20, 22, 28]. Additionally, it has profound social and economic costs, including the burden on healthcare systems, loss of productivity, and societal repercussions like the perpetuation of stigma surrounding mental health, community safety concerns and negative impact on family relationships [9, 20]. Moreover, it poses harm to both self and others, and its associated complications, such as psychological trauma among the victims, suicide, homicide, and pathological bereavement among those who have lost loved ones, may result in further burden to an already fragile mental health system [19]. Therefore, knowledge about factors associated with dual harm enables mental health professionals to develop targeted and effective treatment plans [29]. It facilitates the identification of individuals at higher risk, allowing for tailored interventions and preventive strategies and contributes to breaking down the stigma surrounding mental health issues, fostering a more compassionate and informed societal response [2, 9, 29]. Additionally, understanding the factors influencing dual harm contributes to the development of targeted and evidence-based treatments [29]. This can lead to improved outcomes for individuals experiencing mental health conditions associated with dual harm [29].

Adverse environmental factors like socio-economic disparities, political instability, and limited access to mental health services in Uganda [22, 30] may increase the likelihood of dual harm, rendering it a pressing concern. Moreover, studies examining dual harm in psychiatry in-patient settings, where the expectation that dual harm would be far more prevalent, are scarce [1, 12]. Therefore, the current study examined dual harm prevalence and associated sociodemographic and clinical factors among in-patient individuals in a mental health unit in Uganda.

## Methods

This study was based on a retrospective chart review of the inpatient registered in Health Management Information Systems [HMIS] form 031 of the Mbarara Regional Referral Hospital (MRRH) psychiatry ward from January 2018 to December 2021. Similar data has been used to understand various aspects of Uganda patients [31–35]. The current study was conducted in accordance with the Declaration of Helsinki 2013 and received a formal consent waiver from the Mbarara University Ethical Review Board, reference number MUST-2021-229.

MRRH psychiatry ward is the largest psychiatric facility in southwestern Uganda, with four stationed psychiatrists, and it’s a teaching center for psychiatry residents, medical students, and nursing students. The diagnoses for mental illness are based on both ICD and or DSM criteria, following historical and observational content from the patient, family, and staff. The facility handles and manages people, especially referrals from all over southwestern Uganda. Information about the patients who are managed at MRRH is summarized in the HMIS. All patients in the HMIS have their records stored at the Department of Psychiatry records offices, and the records are managed by professional records managers. The HMIS form 031 captures the following information: date of admission, patient’s number, name, address, age, anthropometric measurements, education level, occupation, gender, next of kin, investigations, presenting complaints, physical symptoms, diagnosis, comorbidities, and treatment given. Information from this form is used to physically trace the patient's chat, where the current data was extracted.

After retrieving all patient charts between January 2018 and December 2021 (n = 3014), the research team created a database containing sociodemographic and clinical characteristics. However, the following variables were used to answer the current research questions: (i) patient’s number, (ii) year of admission, (iii) age, (iv) gender (male, female), (v) marital status, (vi) level of education, (vii) employment status, (viii) presenting complaint including harm to self, others, and property, (ix) history of mental illness in the family, and (x) diagnosis. All data were entered parallel by two pairs of individuals. In case of any discrepancies, AF resolved them.

### Data analysis

Data were analyzed using STATA version 17.0. Categorical variables were presented with frequencies and percentages. The continuous variable (age) was present with a mean and SD. The normality assumption was assessed using the Shapiro-Wilks test. The Chi-square tests for categorical variables were performed to determine significant differences between individuals with different types of aggression. The Fisher Exact test was performed to determine significant differences between individuals with different types of dual harm. The difference between the ages was assessed using the student’s t-test. Bivariate and multiple logistic regressions were performed to assess the association between harm to others and harm to self and to assess the association between dual harm and social demographics. The significant level was less than 5% for a 95% confidence interval.

## Results

### Patient’s characteristics

A total of 3098 participants were included in this study, and slightly more than half of the participants were male (55.97%). According to marital status, married people were the most represented (45.95%), followed by single (40.90%). Participants with no formal or primary level of education represented 60.78%. The most represented diagnosis was bipolar disorder (37.67%), followed by Schizophrenia and other primary psychosis (33.36%), substance use disorder (12.54%), psychosis secondary to a general medical condition (8.67%), and lastly depression (7.75%). Most participants (62.07%) spent only one week in the ward during their admission.

### Prevalence of aggressive behaviors and dual harm

**Fig. 1:**
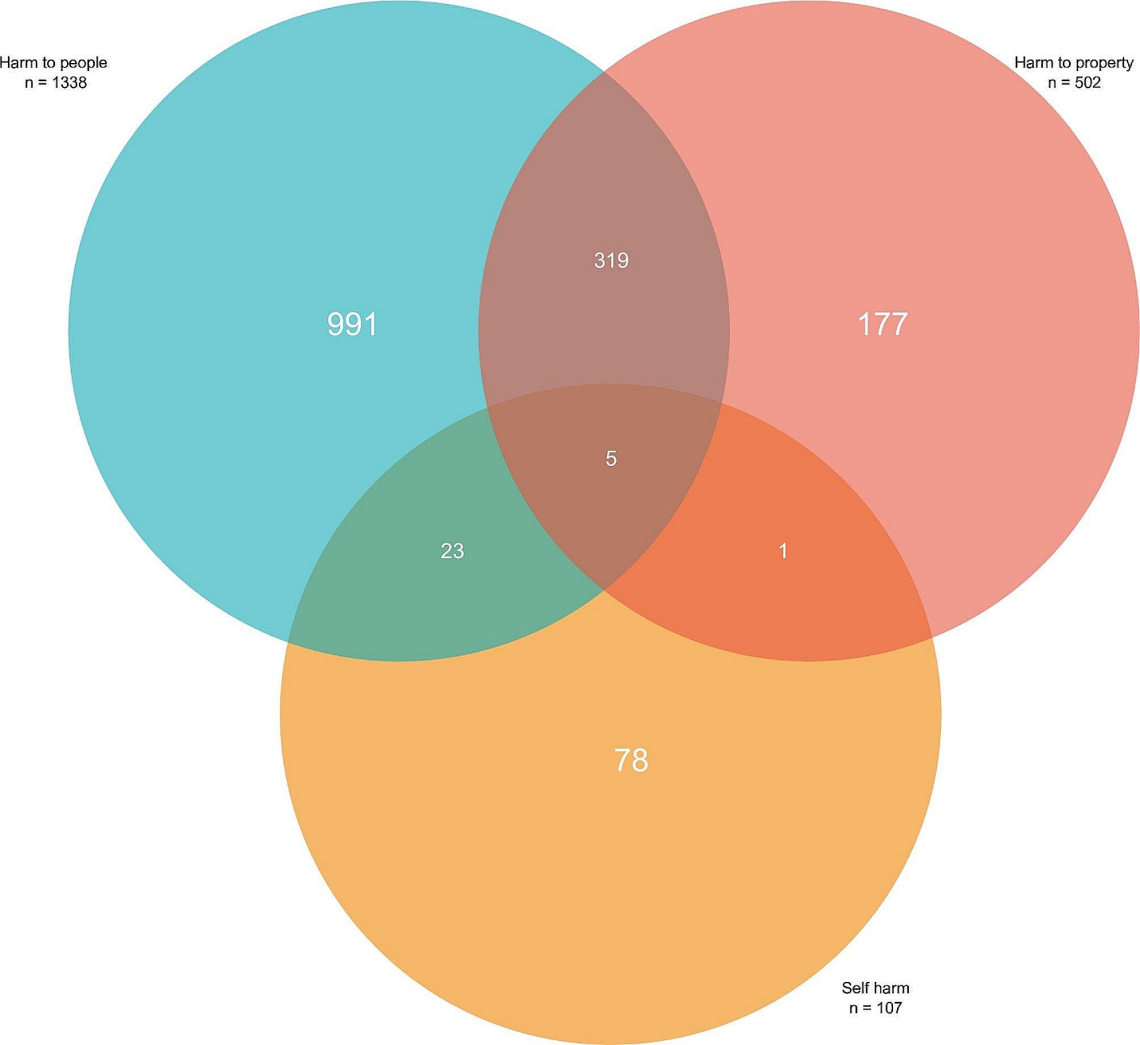
Venn diagram showing the relation between the different aspects of aggression

A total of 29 (1%) individuals experienced dual harm. Five of these had dual harm to both people and property; 23 had dual harm to both others and self; and one individual had dual harm to self and property (see Fig. 1). The individual who had dual harm to self and property was a 49year old divorced male farmer with a negative family history of mental illness.

### Relationship between individual aggressive behaviors type and sociodemographic characteristics

#### Harm to people

Harm to people was statistically more among individuals diagnosed with bipolar disorder (*p*-value < 0.001). On average, those who caused harm to people were younger than those who did not, i.e., mean of 32.27 vs. 34.92, *p*-value < 0.001. More males, 61.53%, caused harm to people than females, 38.47%, *p*-value < 0.001. Harm to people was statistically more among individuals who were single (*p*-value = 0.009) and among those who had been on theward for one week or less compared to those who had been admitted for more than a week (58.51% vs. 41.49, *p*-value = 0.001) (Table 1).

**Table 1:** Relationship between harm to people and sociodemographic characteristics

Variables	Harm		
	To people		
	No	Yes: *n* = 1338 (43.19%)	*P* value
***Age (mean, SD)***	34.92, 14.69	32.27, 12.93)	**< 0.001**
***Sex***			
Female	848, 48.24%	514, 38.47%	**< 0.001**
Male	910, 51.76%	822, 61.53%	
***Marital status***			
Divorced/Separated/widowed	191, 13.40%	139, 12.78%	**0.009**
Single	546, 38.32%	482, 44.30%	
Married/cohabiting	688, 48.28%	467, 42.92%	
***Level of education***			
None	374, 31.30%	266, 29.62%	0.309
Primary	344, 28.79%	288, 32.07%	
Secondary	282, 23.60%	215, 23.94%	
Tertiary	195, 16.32%	129, 14.37%	
***Occupation***			
Unemployed	235, 17.07%	175, 16.43%	0.816
Pupil/Student	198, 14.38%	159, 14.93%	
Farmer	442, 32.10%	357, 33.52%	
Other employment	502, 36.46%	374, 35.12%	
***Diagnosis***			
Bipolar	414, 32.50%	450, 44.12%	**< 0.001**
Schizophrenia	434, 34.07%	331, 32.45%	
Depression	158, 12.40%	20, 1.96%	
Psychosis secondary to GMC*	128, 10.05%	71, 6.96%	
Substance use disorder	140, 10.99%	148, 14.51%	
***Duration on ward***			
*One week*	1,061, 64.73%	739, 58.51%	**0.001**
*Over one week*	578., 35.27%	524, 41.49%	
***Family History of mental illness***			
positive	339, 19.53%	234, 17.66%	0.189
Negative	1,397, 80.47%	1,091, 82.34%	

*General medical condition

#### Harm to property

Harm to property was statistically more among individuals diagnosed with bipolar disorder (*p*-value < 0.001) and those who were single (*p*-value = 0.001). On average, those who caused harm to property were younger than those who did not, i.e., mean of 31.94 vs. 34.41, respectively, *p-*value < 0.001. More males, 65.87%, caused harm to property than females, 34.13%, *p*-value < 0.001. More individuals who had been on the ward for one week (56.41%) caused harm to property than those who had been admitted for more than a week (43.59), *p*-value = 0.006. Harm to property was statistically less among individuals with a positive family history of mental illness than those with a negative family history (22.69% vs. 72.31%, *p*-value = 0.013) (Table 2).

**Table 2:** Relationship between harm to property and sociodemographic characteristics

Variables	Harm		
	To Property		
	No	Yes: *n* = 502 (16.20%)	*P* value
***Age***	34.14, 14.08	31.94, 13.55	**0.001**
***Sex***			
Female	1,191, 45.93%	171, 34.13%	**< 0.001**
Male	1,402, 54.07%	330, 65.87%	
***Marital status***			
Divorced/Separated/widowed	283, 13.55%	47, 11.08%	**0.001**
Single	821, 39.30%	207, 48.82%	
Married/cohabiting	985, 47.15%	170, 40.09%	
***Level of education***			
None	505, 29.74%	135, 34.18%	**0.001**
Primary	496, 29.21%	136, 34.43%	
Secondary	412, 24.26%	85, 21.52%	
Tertiary	285, 16.78%	39, 9.87%	
***Occupation***			
Unemployed	327, 16.10%	83, 20.19%	0.210
Pupil/Student	298, 14.67%	59, 14.36%	
Farmer	666, 32.79%	133, 32.36%	
Other employment	740, 36.44%	136, 33.09%	
***Diagnosis***			
Bipolar	689, 36.09%	175, 45.45%	**< 0.001**
Schizophrenia	635, 33.26%	130, 33.77%	
Depression	171, 8.96%	7, 1.82%	
Psychosis secondary to GMC*	181, 9.48%	18, 4.68%	
Substance use disorder	233, 12.21%	55, 14.29%	
***Duration on ward***			
*One week*	1,536, 63.11%	264, 56.41%	**0.006**
*Over one week*	898, 36.89%	204, 43.59%	
***Family history of mental illness***			
positive	460, 17.95%	113, 22.69%	**0.013**
Negative	2,103, 82.05%	385, 77.31%	

#### Harm to self

Harm to self was statistically more among individuals diagnosed with a diagnosis of depression (*p*-value < 0.001) (Table 3).

**Table 3:** Relationship between self-harm and sociodemographic characteristics

Variables	Harm		
	To Self		
	No	Yes: *n* = 107 (3.45%)	*P* value
***Age***	33.83, 14.05	32.49, 13.06	0.331
***Sex***			
Female	1,319, 44.16%	43, 40.19%	0430
Male	1,668, 55.84%	64, 59.81%	
***Marital status***			
Divorced/Separated/widowed	323, 13.36%	7, 7.37%	0.222
Single	988, 40.86%	40, 42.11%	
Married/cohabiting	107, 45.78%	48, 50.53%	
***Level of education***			
None	621, 30.74%	19, 26.03%	0411
Primary	613, 30.35%	19, 26.03%	
Secondary	474, 23.47%	23, 31.51%	
Tertiary	312, 15.45%	12, 16.44%	
***Occupation***			
Unemployed	394, 16.74%	16, 18.18%	0.630
Pupil/Student	341, 14.49%	16, 18.18%	
Farmer	775, 32.92%	24, 27.27%	
Other employment	844, 35.85%	32, 36.36%	
***Diagnosis***			
Bipolar	852, 38.41%	12, 15.79%	**< 0.001**
Schizophrenia	745, 33.59%	20, 26.32%	
Depression	146, 6.58%	32, 42.11%	
Psychosis secondary to GMC*	198, 8.93%	1, 1.32%	
Substance use disorder	277, 12.49%	11, 14.47%	
***Duration on ward***			
*One week*	1,736, 62.00%	64, 62.75%	0.879
*Over one week*	1,064, 38.00%	38, 37.25%	
***Family History of mental illness***			
positive	551, 18.65%	22, 20.56%	0.614
Negative	2,403, 81.35%	85, 79.44%	

### Factors associated with dual harm

Bivariate and multivariate analysis was done to identify factors associated with dual harm. At bivariate analysis, only male sex was associated with dual harm (odds ratio (OR): 02.49; 95% confidence interval (CI): 01.06–05.85; *p*-value = 0.036). However, there was no factor associated with dual harm at multivariate analysis (Table 4). Even at sensitivity analysis, no factors were identified to be associated with the specific types of dual harm.

**Table 4:** Factors associated with the dual harm

	^Dual harm^			^Bivariate analysis^			^Multivariate analysis^		
	^Absent^	^Present^ ^*n*=29^	^X2 (*p* value)^	^OR^	^95% CI^	^*p*−value^	^AOR^	^95% CI^	^*p*−value^
^**Age**^	^33.82^ ^(14.02)^	^29.59^ ^(13.48)^	^(t value missing) 0.105^	^00.97^	^(00.94–01.01)^	^0.107^	^00.95^	^(00.88–01.03)^	^0.224^
^**Sex**^									
^ Female^	^1,355^ ^44.21%^	^7^ ^24.14%^	^4.70 (0.370)^	^01.00^			^01.00^		
^ Male^	^1,710^ ^55.79%^	^22^ ^75.86%^		^02.49^	^(01.06–05.85)^	^**0.036**^	^01.49^	^(00.45–04.94)^	^0.514^
^**Marital status**^									
^ Divorced/Separated/widowed^	^329^ ^13.23%^	^1^ ^3.70%^	^3.44 (0.199)^	^01.00^			^01.00^		
^ Single^	^1,013^ ^40.75%^	^15^ ^55.56%^		^04.87^	^(00.64–37.02)^	^0.126^	^01.18^	^(00.28–04.99)^	^0.824^
^ Married/cohabiting^	^1,144^ ^46.02%^	^11^ ^40.74%^		^03.16^	^(00.41–24.59)^	^0.271^	^01.00^	^Omitted^	
^**Level of education**^									
^ None^	^637^ ^30.74%^	^3^ ^14.29%^	^2.66 (0.375)^	^01.00^			^01.00^		
^ Primary^	^624^ ^30.12%^	^8^ ^38.10%^		^02.72^	^(00.72–10.31)^	^0.140^	^02.15^	^(00.41–11.40)^	^0.367^
^ Secondary^	^491^ ^23.70%^	^6^ ^28.57%^		^02.59^	^(00.65–10.43)^	^0.179^	^02.23^	^(00.38–12.98)^	^0.373^
^ Tertiary^	^320^ ^15.44%^	^4^ ^19.05%^		^02.65^	^(00.59–11.93)^	^0.203^	^02.24^	^(00.34–14.92)^	^0.404^
^**Occupation**^									
^ Unemployed^	^403^ ^16.66%^	^7^ ^30.43%^	^3.12 (0.411)^	^01.00^			^01.00^		
^ Pupil/Student^	^354^ ^14.63%^	^3^ ^13.04%^		^00.49^	^(00.13–01.90)^	^0.301^	^00.27^	^(00.05–01.29)^	^0.101^
^ Farmer^	^793^ ^32.78%^	^6^ ^26.09%^		^00.44^	^(00.15–01.30)^	^0.138^	^00.15^	^(00.02–01.31)^	^0.085^
^ Other employment^	^869^ ^35.92%^	^7^ ^30.43%^		^00.46^	^(00.16–01.33)^	^0.153^	^00.55^	^(00.15–02.04)^	^0.373^
^**Diagnosis**^									
^ Substance use disorder^	^283^ ^12.47%^	^5^ ^20.83%^	^3.06 (0.505)^	^01.00^			^01.00^		
^ Schizophrenia^	^757^ ^33.35%^	^8^ ^33.33%^		^00.60^	^(00.19–01.84)^	^0.371^	^00.50^	^(00.12–02.14)^	^0.352^
^ Depression^	^175^ ^7.71%^	^3^ ^12.50%^		^00.97^	^(00.23–04.11)^	^0.967^	^00.67^	^(00.06–07.34)^	^0.746^
^ Secondary^	^198^ ^8.72%^	^1^ ^4.17%^		^00.29^	^(00.03–02.47)^	^0.255^	^00.67^	^(00.06–07.37)^	^0.745^
^ Bipolar^	^857^ ^37.75%^	^7^ ^29.17%^		^00.46^	^(00.15–01.47)^	^0.191^	^00.42^	^(00.09–01.85)^	^0.250^
^**Weeks in ward**^									
^ one Week^	^1,786^ ^62.14%^	^14^ ^50.00%^	^1.74 (0.244)^	^01.00^			^01.00^		
^ Over one week^	^1,088^ ^37.86%^	^14^ ^50.00%^		^01.64^	^(00.78–03.46)^	^0.192^	^01.91^	^(00.69–05.30)^	^0.215^
^**History of stressors**^									
^ No^	^568^ ^18.73%^	^5^ ^17.24%^	^0.04 (1.00)^	^01.00^			^01.00^		
^ Yes^	^2,464^ ^81.27%^	^24^ ^82.76%^		^01.11^	^(00.42–02.91)^	^0.838^	^01.70^	^(00.59–04.93)^	^0.328^

## Discussion

This study aimed to investigate dual harm and its related factors among individuals admitted with mental illness. Results showed that the prevalence of dual-harm among these individuals admitted at MRRH was approximately 1%, which is closer to that of 2% reported from hospital records of general psychiatry inpatients in USA [5]. The prevalence reported in this study is much lower than that of 17% reported among general psychiatry outpatients in UK [36], and that of 20% among inpatients in Netherlands [7], and in Finland [37]. The higher prevalence is likely because the study in UK assessed for prior history of violence among those who had attempted suicide and the study in Netherlands used tools to assess for aggression and self-harm while this study denoted aggression reported at admission. Another possible reason for the higher prevalence in the study in Finland is that it was carried out for a longer period (16years) and it focused on homicide-suicide dual harm while the current study looked at non-homicide-suicide dual harm. The prevalence in this study is also much lower than that reported among forensic psychiatry populations [10, 38, 39] likely because forensic populations often consist of individuals who may exhibit complex psychopathological conditions, which can involve a combination of traits, such as impulsivity, emotional dysregulation, and interpersonal difficulties [40]. Additionally, cultural norms and values in Uganda may prioritize communal and family support more [41], potentially resulting in lower rates of dual harm. The robust family networks in Uganda may offer greater care and supervision to individuals with mental disorders [42–44], thus reducing the likelihood of dual harm. In addition, cultural norms in Uganda play a significant role in shaping the behavior and help-seeking patterns of psychiatry patients, often discouraging the presentation of violence in healthcare settings. Family members of violent people with mental illness may be reluctant to seek help at hospitals or clinics because of Africa’s societal norms favoring restraint, which includes physically restraint of the mentally ill, and social cohesion [45, 46]. This is because they may be more worried about maintaining their social connections and reputation [46]. There may also be reporting bias as a result of these cultural norms, when people believe they shouldn’t expose their patients’ violent occurrences to the public since doing so would make them appear terrible [47, 48]. Lastly, aggression is a multifaceted behavior that encompasses various forms, and it remains unclear whether the inpatient charts of psychiatry patients comprehensively captured all forms of aggression like verbal aggression. However, the culture of Ugandan people is changing [49] and the trends may also increase with time.

This study found that being male was significantly associated with dual harm. This finding echoes findings in other studies [11, 50]. This is likely because males may be more likely to engage in risky or dangerous behaviors potentially leading to dual harm [51]. Males resort to aggressive methods in order to ascertain their masculinity, potentially leading to their involvement in dual harm [52, 53]. Additionally, there are higher rates of substance abuse among men, such as alcohol or illicit drugs, can increase the likelihood of aggressive or violent behavior, both toward oneself and others [54–56]. Men might have smaller social support networks or feel less comfortable discussing their mental health issues with others [57], which can lead to increased isolation and a higher risk of harm. Despite the identified relationship at bivariate regression, it was not significant in the multivariate model, an indication of influence of other factors in its relationship with dual harm. An aspect that may be due to the limitation of the present study.

In this study, no specific mental disorder was associated with dual harm. This finding aligns with finding of a systematic review from twenty-seven studies that there is no sufficient evidence that any of the mental illnesses is associated with dual-harm when compared [10]. This implies that various mental disorders are not unique to dual harm, but rather driven by the separate self-harm or aggressive behaviors [10].

Contrary to various studies where prior adverse events resulted in dual harm [23, 24], this study’s findings do not report any association between history of a stressor and dual harm. This is likely because Uganda has a unique cultural context, and individuals may have different coping mechanisms and ways of expressing psychological distress. Additionally, social support [58] or individual resilience [59], could have influenced the outcomes. Furthermore, the way in which stressors were measured and reported in the study could introduce biases i.e., stressors were self-reported, and participants may underreport or fail to recognize certain stressors. In addition, the study might not have captured the delayed effects of long-term stressors.

The absence of identified factors associated with dual harm in the final model of this study can be attributed to the complexities and multifaceted nature of mental health outcomes. Several variables may influence the occurrence of dual harm, but they were difficult to capture comprehensively within the confines of this study as reported findings from history captured at admission. Furthermore, identifying predictors of dual harm might be difficult due to characteristics including individual resilience, distinct coping techniques, and subtle manifestations of discomfort among Ugandan patients. Moreover, the model’s capacity to identify meaningful correlations may be impacted by methodological constraints like the duration taken into consideration and the absence of measuring instruments. The interaction of these variables, together with the variety of psychiatric disorders and personal experiences, highlights the need for more thorough and situation-specific study to gain a deeper understanding of the elements causing twofold harm among Ugandan psychiatric in-patients.

Overall, the study’s finding that 1% of psychiatric in-patients in Uganda experience dual harm, has significant ramifications for mental health services and research in the area. Recognizing dual arm can help mental health providers create sensitive treatment plans that meet the difficulties the patients encounter. These plans may include strengthening coping skills and decreasing aggressive behavior. These results further highlight the importance of increasing mental health service accessibility and increasing dual harm awareness in order to enhance early detection and intervention.

### Limitations and recommendations

The following limitations should be taken into consideration when interpreting the results of this study. Firstly, it should be noted that this was a retrospective evaluation, and study designs like these frequently have missing data. In a study of this nature, the limitations of missing data are universally recognized. We recommend future researchers to use several assessment techniques and employ prospective study designs to ensure accurate detection of the various aspects of dual harm. Secondly, clinical judgment was used for assessment and categorization of the harm because the nature of the harm was documented by the doctors in the patient’s charts. The reported prevalence of dual harm among psychiatry in-patients may not be accurate or consistent due to subjectivity reporting by the caregivers. In order to prevent this bias, future investigators can employ formal techniques and operational criteria for dual-harm aspects, i.e., violence and self-harm. Thirdly, many variables that could have influenced the findings, such as adherence to medication, adverse childhood events, family environment were not retrieved from the records of patients and hence not included in the analysis. We recommend that prospective study designs be used in subsequent research to obtain this kind of data. Furthermore, the study population is limited to in-patients of general psychiatry and excludes members of the forensic psychiatry population because the psychiatric unit is not gazetted to house forensic patents, who frequently face unique clinical and legal circumstances that put them at a higher risk of harm. Hence, the lack of this particular patient category in our research may restrict the generalization of our conclusions to a larger psychiatric community and may understate the general prevalence of dual harm among inpatient psychiatric patients.

## Conclusion

Dual harm is not a common phenomenon documented among inpatients with mental illness at a tertiary facility in Uganda. According to our research findings, no factors were found to be statistically linked with dual harm among psychiatry in-patients. This indicates that dual harm in this community is complicated and varied, highlighting the need for more research and sophisticated methods to comprehend the variables influencing such occurrences. Dual harm is still less explored and further research is need in completely understand this phenomenon in Uganda and Africa at large.

## Data Availability

The datasets will be made available to appropriate academic parties on request from the corresponding author.
